# Successful management of pregnancy in an African woman with Klippel Trenaunay syndrome

**DOI:** 10.11604/pamj.2013.16.99.3395

**Published:** 2013-11-16

**Authors:** Jean Dupont Ngowa Kemfang, Walter Pisoh Dobgima, Regine Mbouopda Motzebo, Anny Ngassam, Marcus Fokou, Jean-Marie Kasia

**Affiliations:** 1 Obstetrics and Gynecology Unit, Yaounde General Hospital, P.O. Box 5408, Yaounde, Cameroon; 2 Department of Obstetrics and Gynecology, Faculty of Medicine and Biomedical Sciences, University of Yaounde I, P.O. Box 1364, Yaounde, Cameroon; 3 Anesthesiology Unit, Yaounde General Hospital, P.O. Box 5408, Yaounde, Cameroon; 4 Surgery Unit, Yaounde General Hospital, P.O. Box 5408, Yaounde, Cameroon

**Keywords:** Klippel Trenaunay syndrome, pregnancy, Yaounde

## Abstract

Klippel Trenaunay syndrome (KTS) is a rare congenital disease characterized by a triad of cutaneous hemangioma, varicose veins and bone or soft tissue hypertrophy. Cases of pregnancy complicated by KTS are rare. There is an increased risk of thrombo-embolic disease and hemorrhage during pregnancy. Both obstetric and anesthetic management of KTS in pregnancy can be rather complicated. We present a successful management of pregnancy in an African woman with KTS at Yaounde General Hospital, Cameroon.

## Introduction

Originally describe in 1900, Klippel Trenaunay syndrome (KTS) is a rare congenital disease characterized by a triad of extensive cutaneous hemangiomas, venous varicosities and soft tissue or bone hypertrophy affecting a lower limb and/or arm on one side [1]. The cases of pregnancy complicated by KTS are rare [2, 3]. The normal physiological changes of pregnancy such as increase in venous pressure, leg edema, venous stasis and increased cardiac output, exacerbate the problems of this syndrome resulting in an increased risk of thrombo-embolic disease and hemorrhage [3]. Both obstetric and anesthetic management of KTS in pregnancy can be rather complicated [3]. We report a case of successful management of pregnancy complicated by KTS at the Yaounde General Hospital, Cameroon.

## Patient and observation

We present a case of a 39 years old African woman, G2P1011. She had varicose veins and swelling on the left lower limb since the age of 3 years. She was diagnosed of KTS at the age of 29 years for which varicectomy had been done 7 times without success. She had a polymyomatous uterus and no family history of KTS. She was referred to our hospital at 16 weeks of pregnancy for a termination of her pregnancy because she presented a KTS. Considering her strong desire to have a child, the decision for continuation of the pregnancy was made by a multidisciplinary staff (obstetrician, anesthesiologist, vascular surgeon and hematologist). She did not receive any anticoagulants during her routine antenatal care. The course of the pregnancy was uneventful. On the basis of large multiple uterine myomas, we decided to deliver her baby by cesarean section.

**Figure 1:**
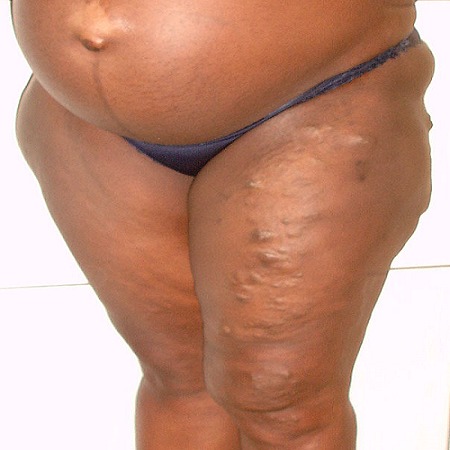
View of venous varicosities and prominent hypertrophy on the left thighs during pregnancy

**Figure 2:**
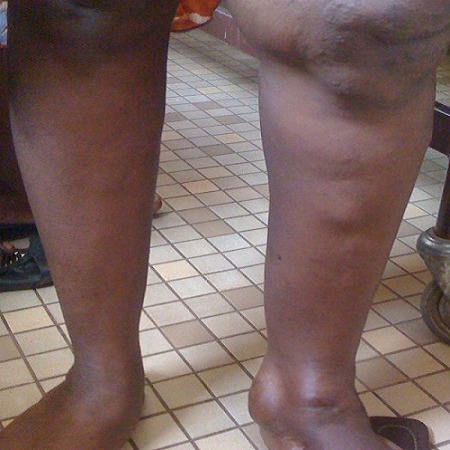
View of venous varicosities and prominent hypertrophy on the left leg during pregnancy

**Figure 3:**
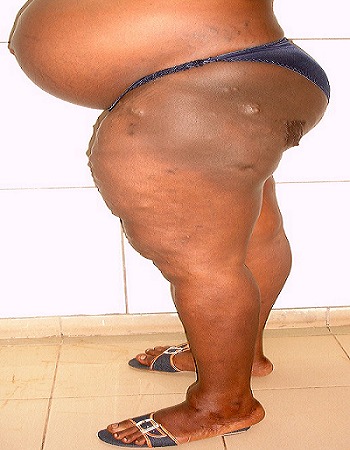
Lateral view of the left lower limb during pregnancy

On admission at 36weeks of gestation, physical examination revealed prominent hypertrophy and multiple venous varicosities on the left lower limb (Figure 1, Figure 2, Figure 3). Laboratory studies revealed a normal coagulation profile. At 37 weeks of gestation, she underwent a cesarean delivery by a Pfannenstiel incision under spinal anesthesia. No abnormal vessels were noted around the polymyomatous uterus during the surgery. A female baby weighing 3300g was delivered, with APGAR scores of 9/10 and 10/10 at the 1st and 5th minutes respectively. No abnormal finding indicative of neonatal KTS was observed. The established blood loss was about 600cc and no blood transfusion was required. Elastic compressive stockings and low-molecular-weight heparin was administered for 7 post operative days. The post operative course was uneventful and she was discharged with her healthy baby.

## Discussion

Klippel-Trenaunay syndrome was first described in 1900 by two French physicians, Maurice Klippel and Paul Trenaunay. They called the disease naevus vasculosus steohypertrophicus[1]. KTS is defined by the presence of a combined vascular malformation of the capillaries, veins, and lymphatics with congenital venous abnormalities and limb hypertrophy [4]. Our patient was diagnosed of KTS at the age of 29years.

The etiology of KTS is unknown. Several theories have been proposed including abnormalities of the sympathetic nervous system resulting in dilatation of the arteriovenous anastomoses or obstruction of the deep veins and persistence of fetal microscopic small arterioveneous anastomoses [5]. In about 1% of cases of Klippel-Trenaunay syndrome, a genetic pattern has been described, but the gene has not yet been identified [4–6]. Our patient had no family history of KTS and probably, she had a low risk of genetic etiology.

KTS in pregnant women is extremely rare, and a few cases have been published in literature [2, 3, 6]. Our patient was the only case of KTS with pregnancy documented during the 25 years of existence of our hospital, confirming the rarity of this disease. Prenatal sonographic findings in a case of KTS include fetal ascites and subcutaneous cystic lesions associated with a relatively low level of maternal serum alpha-fetoprotein and a relatively high level of maternal serum beta-human chorionic gonadotrophin. [7]

Patients with KTS have an increased risk of thromboembolic disease. Stein et al. [8] and Fait et al. [9] reported respectively, cases of right lower leg deep vein thrombosis and left calf deep vein thrombosis during pregnancy. Anticoagulant therapy with aspirin or heparin calcium during pregnancy could effectively prevent thromboembolic disease [2, 8, 9]. In our case, the elastic compressive stockings and prophylactic low-molecular-weight heparin were started only after delivery as the patient was considered at high risk for postpartum thrombosis due to the varicosities. The presence of neuraxial vascular anomalies can complicate spinal anaesthesia. It is advised to perform magnetic resonance imagining detecting angiodysplastic vascular structures next to the spinal cord to avoid in these cases a traumatic puncture of these vessels [2, 10]. A spinal anaesthesia was done in our case without any complication. Magnetic resonance imaging was not done in our case because it is inexistent in our setting.

## Conclusion

Pregnancy complicated by KTS is not an indication for termination of pregnancy. Our patient completed her pregnancy without any complications and delivered a healthy baby. A multidisciplinary approach with the obstetrician, anesthesiologist, vascular surgeon and haematologist forms the mainstay of the management of these patients. The use of prophylactic anticoagulants is generally advised during the pregnancy and postpartum period.
